# Tripartite motif-containing protein 50 suppresses triple-negative breast cancer progression by regulating the epithelial–mesenchymal transition

**DOI:** 10.1080/15384047.2024.2427410

**Published:** 2024-11-13

**Authors:** Danxiang Chen, Jing Jiang, Wei Zhang, Xinlin Li, Qidong Ge, Xia Liu, Xujun Li

**Affiliations:** a Department of Oncology, Ningbo No. 2 Hospital, Ningbo, Zhejiang, PR China; b Department of Breast Surgery, Ningbo No. 2 Hospital, Ningbo, Zhejiang, China; c Department of Anesthesiology, Ningbo 1st Hospital, Ningbo, Zhejiang, China

**Keywords:** TRIM50, EMT, triple-negative breast cancer, biomarker

## Abstract

**Background and Objectives:**

Tripartite motif-containing protein 50 (TRIM50) is a recently discovered E3 ubiquitin ligase that participates in tumor progression. TRIM50 is overexpressed in many cancers, although few studies focused on TRIM50‘s role in breast cancer.

**Methods:**

We overexpressed TRIM50 in triple-negative breast cancer cell lines using plasmid and found that TRIM50 upregulation markedly reduced breast cancer cell proliferation, clone formation, and migration, as well as promoted breast cancer cell apoptosis. Western blotting revealed that accumulated TRIM50 resulted in both mRNA and protein depletion of SNAI1, and partially attenuated the epithelial–mesenchymal transition (EMT) induced by SNAI1.

**Results:**

In this study, we demonstrate that TRIM50 is downregulated in human breast cancer and that its overexpression closely correlates with diminished invasion capacity in breast cancer, suggesting that TRIM50 may serve as a diagnostic marker and therapeutic target.

**Conclusion:**

TRIM50 plays a key role in breast cancer proliferation and potentially serves as a prognostic and therapeutic target.

## GRAPHICAL ABSTRACT

**Figure:**
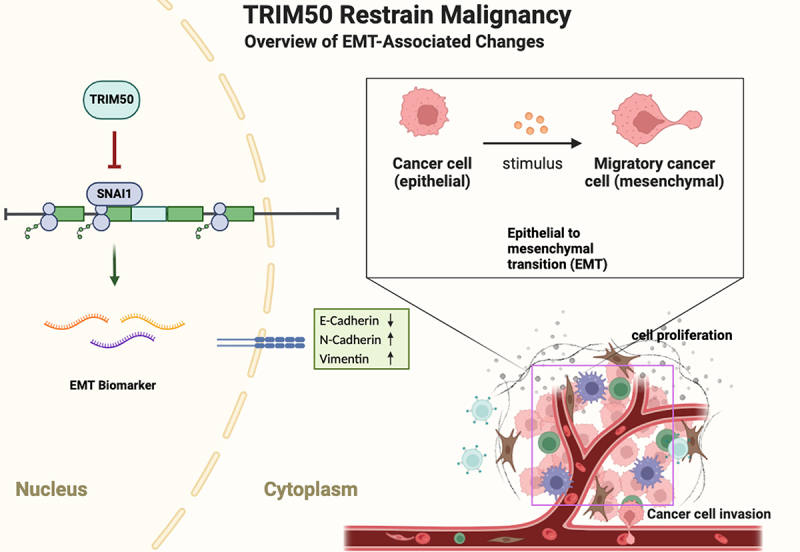

## 1. Introduction

For the past decades, global incidences of breast cancer (BC) have reached 2.3 million cases estimated in the year 2020, gradually outstripping lung carcinoma in morbidity with 11.4% being recorded.^1^ The domestic incidences of breast cancer in China reportedly climbed in numbers to 429,105 cases per year in 2022, accompanied by 124,002 BC-specific death cases.^2^ BCs are characterized by inner genetic heterogeneity, which typically fall into the following three categories: Luminal subtype, human epidermal growth factor receptor 2 (HER2)-positive subtype, and triple-negative breast cancer (TNBC).^3^ Afflicted with the absence of intervention targets and confined by chemotherapy alone, TNBCs only making up a 15–20% proportion of all BC cases tend to be profiled as more clinically invasive and earn more substantial scientific interests^4^ therein, which elucidates the significance of unveiling the carcinogenesis mechanism behind TNBC.^5–7^

Protein ubiquitination denotes a post-translational modification of proteins in charge of cell cycle regulation, genome integrity, cell death, inflammatory signal transduction, and pathogen defense.^8–12^ The activation, binding, and connection of ubiquitination of proteins are performed by the E1 ubiquitin-activating enzyme, E2 ubiquitin-binding enzyme, and E3 ubiquitin ligase.^13^ TRIM family is a classical E3 ubiquitin ligase responsible for transferring ubiquitin from the E2 ubiquitin-binding enzyme to the target protein in the last step of the ubiquitin cycle.^14^ TRIMs functioned in multiple biological activities like proliferation, apoptosis, metastasis, and ferroptosis in various cancers.^15–18^ Besides, TRIM could strengthen autophagy process depending on its K63-linked poly-ubiquitination,^19^ which is deemed as another proteolytic pathway for intracellular protein degradation, and TRIM proteins may help to delineate the interaction between the two proteolytic systems. Remarkably, emerging TRIMbody-Away Technique constructed on TRIM21 utilized both ubiquitin-proteasome and autophagy-lysosome pathways to target intracellular protein and foster a potential application of degrader technologies.^20,21^

It has been found that TRIM59 stabilizes PDCD10 by inhibiting RNFT1-induced K63 ubiquitination and subsequent p62 selective autophagic degradation, thus promoting the deformation, movement, and invasive ability of breast cancer cells.^22^ Suzuki and other scholars found that TRIM25 expression suggested the poor prognosis of breast cancer patients and was related to breast cancer subtypes, and in the ERα, the expression was significantly increased in negative basal cells like breast cancer.^23^ TRIM25 has been proven to act as an E3 ligase to recruit E2 UBCH8 ubiquitinating 14-3-3σ protein and subsequent 14-3-3σ protein degradation in its proteasome, thereby downregulating G2 cell cycle arrest-related 14-3-3σ protein and promoting carcinogenesis of breast cancer.^24^

We interrogated the differentially expressed genes in the Gene Expression Omnibus (GEO) database and discovered a novel TRIM member, namely TRIM50, which is down-regulated in breast cancer. Evidenced by previous research in gastric cancer, pancreatic cancer, and hepatocellular carcinoma, we discern that TRIM50 predominantly performed as a tumor suppressor gene via dampening the output of epithelial–mesenchymal transition (EMT)-related pathway.^25–27^ However, the role of TRIM50 in breast cancer and its underlying molecular mechanism have not been reported yet and merits further dissection.

Herein, our present study anatomized the clinical value of TRIM50 in breast cancer by bioinformatics, cell experiments, and in vivo xenograft experiments. We steadily probed into TRIM50 expression’s association with malignancy behavior of TNBC, specific anti-tumor mechanism, as well as interaction protein in TNBC. In this regard, this study put forth a promising target for TNBC and generated fresh insight into the therapy of TNBC.

## 2. Material and methods

### 2.1. Bioinformatics

The expression of TRIM50 at the mRNA level was analyzed using the microarray gene expression data sets obtained from the Gene Expression Omnibus (GEO) database (http://www.ncbi.nlm.nih.gov/geo/) in the National Center for Bioinformatics analysis (NCBI) with the accession codes GSE109169 (https://www.ncbi.nlm.nih.gov/geo/query/acc.cgi?acc=GSE109169), GSE86374 (https://www.ncbi.nlm.nih.gov/geo/query/acc.cgi?acc=GSE86374), GSE162228 (https://www.ncbi.nlm.nih.gov/geo/query/acc.cgi?acc=GSE16228) and GSE37751 (https://www.ncbi.nlm.nih.gov/geo/query/acc.cgi?acc=GSE37751). GSE109169 microarray (GPL5175) included 25 BC tissues and 25 adjacent nontumor tissues.^28^ GSE86374 microarray (GPL6244) included 124 BC tissues and 35 adjacent nontumor tissues. GSE162228 microarray (GPL570) included 24 BC tissues and 109 adjacent nontumor tissues.^29^ GSE37751 microarray included 61 BC tissues and 47 adjacent nontumor tissues.^30–32^

### 2.2. Clinical characteristics and tissue samples

Clinical samples were obtained from 45 Asian female patients who underwent breast-conserving surgery or mammectomy from April 2020 to October 2020 at Ningbo 2nd Hospital (Ningbo, China). Clinical candidates were histopathologically proven as primary breast carcinoma, with those experiencing preoperative biopsy, chemotherapy, or radiotherapy excluded. The tissue specimens were immediately snap-frozen in frozen pipes pre-cooled at 4°C and followed in −80°C liquid nitrogen for lasting preservation. Fresh tumor and adjacent non-neoplastic tissues acquired intraoperatively were split into two pieces: paraffin-embedded tissues were fixed in paraformaldehyde for immunohistochemical staining, while remainders were homogenized to extract RNA and detect RNA level by RT-qPCR. Complete clinicopathological records were mandatory, including gender, age, pathological tumor size and lymph status, estrogen receptor (ER), and human epidermal growth factor receptor-2 (HER2). Tumor staging was determined by the Eighth Edition of the Cancer Staging Manual by the American Joint Committee on Cancer (AJCC). All patients or their legal representatives were fully informed and consented. This study involving human experiments adhered to the Declaration of Helsinki principles and was authorized by Ningbo 2nd Hospital institutional review board (approval No. wydw2020-0857).

### 2.3. Antibodies

Rabbit polyclonal antibodies against TRIM50 for IHC (#ab272586) and WB (#NBP2 -85,995) were purchased from Abcam and Novus Biologicals, respectively. Rabbit polyclonal antibodies against SNAI1 (#26183-1-AP), E-cadherin (#20874-1-AP), N-cadherin (#22018-1-AP), vimentin (#10366-1-AP), GAPDH (#10494-1-AP) for WB were purchased from Proteintech Technology, USA. Horseradish peroxidase (HRP)-linked secondary antibody for WB was purchased from Shanghai Beyotime Institute of Biotechnology. The manufacturers and catalog numbers of involved reagents, chemicals, and antibodies were kept on record in Supplement Table SI.

### 2.4. Immunohistochemistry analysis

The breast carcinoma and the corresponding para-carcinoma tissues collected at the local cohort were embedded as paraffin blocks and subsequently sliced up as preconditioned 4 μm-thick tissue sections. Immunohistochemical antibody against TRIM50 was accessible by Abcam (#ab272586, 1:100 dilution). Before commencing with the IHC staining protocol, sections experience deparaffinization with xylene, rehydration with a gradient ethanol series, and thermal-treated (121°C for 10 min) antigen retrieval in 10 mmol sodium citrate buffer (pH 6.0). Then sections were incubated with primary antibody against TRIM50 at 4°C overnight and colored by an HRP-labeled secondary antibody and diaminobenzidine (DAB) color development Kit (Beyotime, Shanghai, China) at 37°C for 1 h. The sections were finally redyed with hematoxylin, dehydrated, cleared, and encapsulated. TRIM50 protein expression levels were quantified by immunostaining intensity as the proportion of positive cells.

### 2.5. RNA extraction and real-time quantitative polymerase chain reaction

RNA was manually isolated from cultured cell lines and collected tissues by Trizol reagent (Invitrogen, Waltham, MA, USA). The purity of the extracted RNA was measured by a NanoDrop™ spectrophotometer (USA). The OD260/OD280 ratio indicates the purity of the extracted RNA. Then isolated RNA was diluted into a volume of 14 μL and heated at 70°C for initial denaturation. Single-stranded cDNA was synthesized in the reverse transcription reaction system based on the protocol of Revertra Ace qPCR RT Kit (Toyobo, Osaka, Japan): total RNAs of 14 μL initially denaturated at PCR amplifier (Bio-rad, USA), together with 1 μL mixture of oligo(dT) and random primer, 1 μL reverse transcriptase, and 4 μL RT buffer. Besides, the THUNDERBIRD®SYBR® qPCR Master Mix kit (Toyobo, Osaka, Japan) was used to configure the PCR reaction system, which consisted of 200 ng/μl cDNA, 0.8 μl forward and reverse primers, and SYBR reagent with a final volume of 20 μl. The 7500 real-time PCR system (ABI) was conducted for real-time PCR analysis with the starting step of enzyme stimulation at 95◦C for 2 min, followed by 40 cycles of denaturation at 95◦C for 10 seconds, annealing at 55◦C for 15 seconds, and extension at 60◦C for 1 min. Relative RNA levels were quantified by ΔΔCt method and normalized to household gene (GAPDH). PCR experiments were performed in triplicate. Sequences of primers for TRIM50 and GAPDH are detailed as follows: TRIM50-F: 5’-CCCATTTGCCTGGAGGTCTTC-3’, TRIM50-R: 5’-CAGGACAGCATAGCTCGGAG-3’. GAPDH-F: 5’-CATGAGAAGTATGACAACAGCCT-3’, GAPDH-R:5’-AGTCCTTCCACGATACCAAAGT-3’.

### 2.6. Cell culture

Human mammary epithelial cells (MCF-10A) and human TNBC (MDA-MB-231, BT549, and Hs-578T) cells were supplied by Shanghai Cell Biology, Institute of the Chinese Academy of Sciences (Shanghai, China). MDA-MB-231、Hs-578T cell lines were cultured in DMEM (Gibco, USA) containing 10% fetal bovine serum (FBS) and 1% penicillin-streptomycin in a standard culture condition at 37°C, 95% air, and 5% CO2; BT549 cell line was in RPMI-1640 (Gibco, USA) supplemented with 10% FBS and 1% penicillin-streptomycin; and MCF-10A cell line was in MCF-10A special culture medium (Procell, Wuhan, China).

### 2.7. Western blotting

Three TNBC cell lines simultaneously transfected with pcDNA3.1-TRIM50 and pcDNA3.1-vector plasmid were seeded in a six-well plate. Protein lysis buffer blended with radioimmunoprecipitation (RIPA) lysis buffer and phenylmethanesulfonyl fluoride (PMSF) at a ratio of 1:100 were applied to lyse cells and extract total proteins. Standard operating fluid based on the manufacturer’s instruction of bicinchoninic acid (BCA) assay (Solarbio, Beijing, China) was used to form standard protein concentration plotting and quantify the sample protein concentration as a reference. Extracted proteins were separated by SDS-polyacrylamide gel electrophoresis (SDS-PAGE) and blotted on polyvinylidene difluoride (PVDF) membrane, and encapsulated in 5% skimmed milk. Various proteins were probed with primary antibodies against TRIM50 (#NBP285995, 1:200 dilution, Novus Biologicals, USA), E-cadherin (#20874-1-AP, 1:5000 dilution, Proteintech, USA), N-cadherin (#22018-1-AP, 1:2000 dilution, Proteintech, USA), vimentin (#10366-1-AP, 1:2000 dilution, Proteintech, USA), GAPDH (#10494-1-AP, 1:5000 dilution, Proteintech, USA), Snail (#26183-1-AP, 1:1000 dilution, Proteintech, USA) at 4°C overnight, and subsequent second antibodies. Hereafter, blots were developed by enhanced chemiluminescent (ECL) reagents (NCM Biotech, Suzhou, China) and analyzed by ImageJ software (National Institutes of Health).

### 2.8. Plasmid

PcDNA3.1-vector and pcDNA3.1-TRIM50 plasmid were designed and synthesized by Boyun Biotech Co. Ltd. (Shanghai, China). The pcDNA3.1-TRIM50 plasmid that targeted the TRIM50 sequence was utilized for the transfection and amplification of the target gene using LipofectamineTM RNAiMAX reagent (Invitrogen, Carlsbad, USA). Cells were collected at 1 week after transfection. Cells transfected with pcDNA3.1-vector served as negative control. RT-qPCR and Western blotting were carried out to detect transfection efficiency.

### 2.9. Proliferation, colony formation, and wound-healing assays

Cells transfected with pcDNA3.1-vector and pcDNA3.1-TRIM50 plasmid were cultured in 96-well plates with 3 × 10^3^ cells per well. The proliferation ability was first determined by cell counting kit-8 (CCK8) (Beyotime, Shanghai, China). After the CCK8 reagent was added to 96-well plates at 10 μL per well and incubated at 37°C for 3 h, the optical density (OD) absorbance at 450 nm was measured in the microplate reader to calculate proliferation times. Then, the abovementioned cells were planked on the 6-well plate with 1000 cells per well at 37°C for 8 days to form colonies, and 2 mL 0.1% crystal violet for dyeing was added to visual counting. To assess mobility, the wound-healing assay was performed. The adherent cell monolayer at the bottom was scratched using a 20-μl pipette tip delicately to mimic a wound zone. The initial wound healing image was snapped using the 40X optical microscope, along with 24 h imaging to compare recovery capacity. The abovementioned experiments were performed in quadruplicate.

### 2.10. Cell migration and matrigel invasion assays

Cells transfected with pcDNA3.1-vector and pcDNA3.1-TRIM50 plasmid were cultured in 6-well plates with 5 × 10^6^ cells per well, suspended in a serum-free medium. And 6.5-mm transwell inserts with 8 μm polycarbonate membrane (Corning, USA) were inoculated with 5 × 10^4^ cells per well to perform migration analysis. In synchronism, the same procedure was conducted to perform invasion analysis with inserts precoated with Matrigel (Corning, USA). Serum-free medium was added to the upper chamber, while medium with 10% FBS was added to the lower chamber, which induced chemotactic migration from the upper toward the lower chamber. After incubation for 24 h at 37°C, invasive cells getting through the polycarbonate membrane were fixed in 4% paraformaldehyde for 30 min, stained with crystal violet and subsequently counted under an optical microscope, while noninvasive cells were removed using cotton swabs.

### 2.11. Flow cytometry for cell apoptosis

For the apoptosis assay, cells transfected with pcDNA3.1-vector or pcDNA3.1-TRIM50 plasmid for 48 h were collected and then incubated with Annexin V-FITC (Solarbio, Beijing, China) and propidium iodide (PI) for 15 min. Phosphatidylserine (PS) originally anchored at cytoplasmic domain could be translocated into the cell membrane in the interim phase of apoptosis, and fluorescence-labeled Annexin V with high affinity for PS served as a detector for early apoptosis. When the apoptotic cells encounter sustained damage and caused an irresversible disruption of the cell membrane in the late phase, nucleic acid dye (such as PI) initially inaccessible to the intracellular surface could enter the cell and produce quantified signals. The quantity of apoptotic cells was determined with a flow cytometer (Biorad, Berkeley, CA, USA).

### 2.12. Immunofluorescence

Transfected cells cultured in 6-well plates were first rinsed with PBS in triplicate, fixed with precooled 4% formaldehyde for 30 min, and then treated with 0.1% Triton for 10 min. After membrane rupture and blockage with 5% BSA, cells were incubated with the primary antibody in the wet box overnight at 4°C, subsequent 2 mg/mL secondary antibody (#ab150073, Abcam, Britain) in a dark place at 37°C for 1 h and counterstained with antifade mounting medium with DAPI (Beyotime, Shanghai, China). Digital images were acquired using an inverted fluorescence microscope (Leica, German).

### 2.13. In vivo xenograft model

Twelve female 4–6-week-old BALB/c nude mice fed in the Animal Center of Ningbo 2nd Hospital were supplied by Shanghai Laboratory Animal Research Institution, Chinese Academy of Sciences (Shanghai, China). All the laboratory mice were maintained according to NIH guidelines for the care of laboratory animals and labeled with ear punch numbered in series. Cells transfected with pcDNA3.1-TRIM50 and pcDNA3.1-vector plasmid (2 × 10^6^ cells in 200 µl DMEM medium) were randomly divided into two groups (*n* = 6). Mice in both groups were injected subcutaneously into the right axillary tissue with cotton pressing puncture point after needle withdrawal. The tumor volume was measured by vernier caliper at an interval of 4 days. After a four-week follow-up observation, the surviving mice carrying visible neoplasias were treated with resection operation after administering inhalation anesthesia and consequently euthanized. The abovementioned animal experiments were approved by the Animal Experimentation Ethics Committee of Ningbo 2nd Hospital (approval No. wydw2021-0063)

### 2.14. Statistical analysis

Each assay was conducted in triplicate. The data of the study were presented as mean ± standard deviation (SD). Variables in accordance with the normal distribution analyzed by the Shapiro–Wilk test would be investigated by Student’s t-test. The abnormally distributed variables would be analyzed by the Mann–Whitney test or Wilcoxon test. Chi-square test was used for analyzing the correlation between TRIM50 expression and clinicopathological features. Comparison between two groups was assessed by Student’s t-test, and one-way ANOVA test was advocated for multi-group comparison. Survival analysis was analyzed via Kaplan Meier analysis and log-rank test. *p* value <0.05 was considered with a statistically significant difference. All statistical analysis was performed using GraphPad Prism version 9.0.0 (GraphPad Software, San Diego, California, USA).

### 2.15. Immune landscape exploration

TISIDB website (http://cis.hku.hk/TISIDB/download.php) collects the immunostimulator, immunoinhibitor, chemokines, and human leukocyte antigen. Difference analysis was performed using Wilcoxon to observe the difference in the expression of immune-related molecules in the high/low expression group of TRIM50, and the average expression is visualized by heat maps. Next, we exploited TIP (Tracking Tumor Immunophenotype), a meta-server that systematically integrates “ssGSEA” and “CIBERSORT”, to analyze and visualize the anti-cancer immune state and the proportion of immune-infiltrating cells in the seven steps of the cancer immune cycle using RNA-seq or microarray data. Spearman correlation between TRIM50 and TIP scores, as well as the autocorrelation between TIP scores were analyzed, and the linkET package was used for visualization. Besides, the immune infiltration data of all TCGA samples are obtained from the TIMER2.0 database. The Spearman correlation coefficients obtained from the analysis are comprehensively visualized with heat maps in order to intuitively understand the relationship between different cell types and gene expression under different algorithms.

## 3. Results

### 3.1. TRIM50 was expressed at a low level in human TNBC tissues and cells

**Figure 1.**
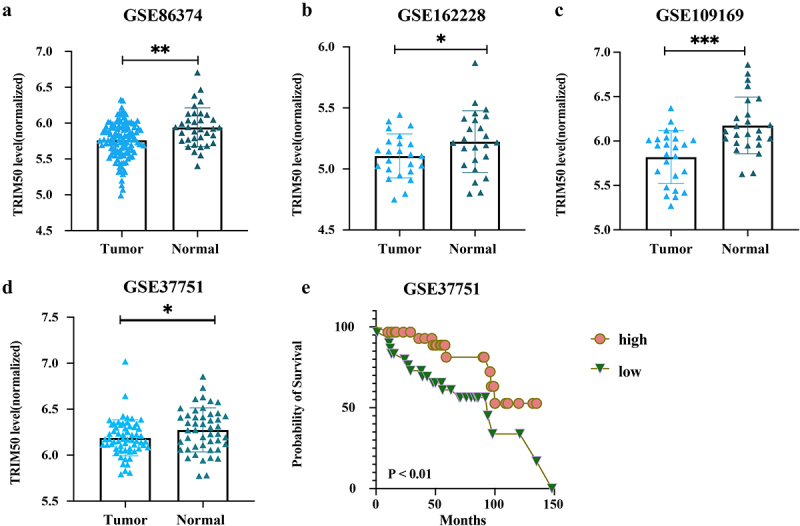
TRIM50 expression in breast cancer and its association with prognosis derived from GEO datasets. GEO datasets (GSE86374, GSE162228, GSE109169, and GSE37751) demonstrated a relationship between TRIM50 levels and BC tumorigenesis. In addition, Kaplan Meier curves show significant association of TRIM50 expression with BC prognosis in the GSE37751 dataset. *p < .05, **p < .01, ***p < .001, ****p < .0001. Each dot represents one sample. Graph bars represent mean ± s.D.

In the present study, we first investigated the public database to present the potential role of TRIM50 in breast cancer. Our presupposition was supported by the differential expression analysis on the GSE109169, GSE86374, GSE37751, and GSE162228 datasets (the detailed information is shown in Table I). Among the overlapping BC-related differentially expressed genes identified from the above-mentioned datasets, TRIM50 served exclusively as a down-regulated gene in comparison with others in the TRIM family. Besides, TRIM50 expression is lower in breast cancer than in normal mammary tissue with a statistically significant difference. Based on the Kaplan – Meier plotter, lower expression of TRIM50 is significantly associated with a lower probability of survival in the GSE37751 dataset (*p* < .01, Figure 1).

**Table I.** The characteristics of enrolled GEO dataset.

Dataset	Author	Year	Platform	Sample (Normal: Tumor)
GSE109169	Chang JW	2018	GPL5175[HuEx-1_0-st] Affymetrix Human Exon 1.0 ST Array	25:25
GSE86374	Rebollar-Vega R	2018	GPL6244[HuGene-1_0-st] Affymetrix Human Gene 1.0 ST Array	35:124
GSE37751	Terununa A	2013	GPL6244[HuGene-1_0-st] Affymetrix Human Gene 1.0 ST Array	47:61
GSE162228	Chen Y	2021	GPL570[HG-U133_Plus_2] Affymetrix Human Genome U133 Plus 2.0 Array	24:109

**Figure 2.**
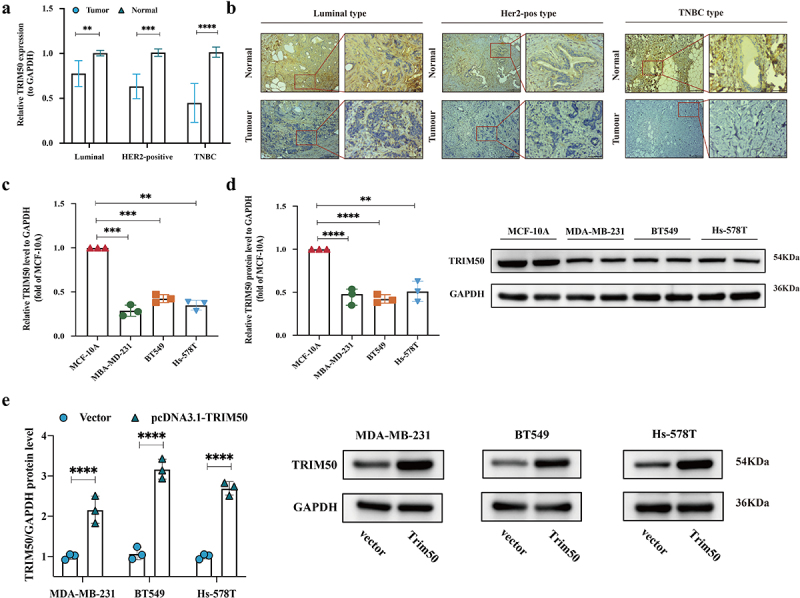
TRIM50 expression is markedly diminished in BC. (a) qRT-pcr analysis of the indicated genes in local cohorts stratified on the basis of BC subtypes (*n* = 31 for luminal condition; *n* = 9 for her-positive condition; *n* = 5 for TNBC condition). (b) Representative images of immunohistochemical (IHC) analysis of 3 breast cancer samples stratified on the normal and tumor conditions (*n* = 5 for each condition). Samples were stained with anti-TRIM50 antibody. (10X scale: 200 µm; 40X scale: 40 µm). (c) qRT-pcr and (d)western blot analysis of indicated proteins in MDA-MB 231, BT549, and Hs-578T cells compared to mammary epithelial cell MCF-10A. Three dots for each cell line represent *n* = 3 biological replicates. (e) The transfection efficiency of pcDNA3.1-TRIM50 plasmids was confirmed by western blotting after these transfected pcDNA3.1-TRIM50 plasmids were screened out by purinomycin. *p < .05, **p < .01, ***p < .001, ****p < .0001. Graph bars represent mean ± s.D.

We next examined the relationship between TRIM50 expression at the tissue level in the local cohort (*n* = 45). Compared with mRNA levels of TRIM50 in 45 pairs of breast cancer, TRIM50 levels in adjacent breast tissues were significantly higher (Luminal:*N* = 31, *p* < .01; Her2:*N* = 9, *p* < .001; TNBC:*N* = 5, *p* < .0001). This association was distinctly observed in the TNBC subgroup, which might predict a biological index of malignancy degree (Figure 2a).

Based on the requirement for the integrity and suitable size of paraffin-embedded tissue, 15 pairs of cancer and normal tissue blocks (5 pairs of Luminal, HER2-pos, TNBC samples in each among) were selected for IHC analysis. Analysis of immunohistochemical staining under optical microscopes revealed that TRIM50 was significantly diminished in luminal, HER2-positive breast cancer, and TNBC than in normal breast tissues. The fact that a higher positive staining (brown) rate was detected in most normal tissues further validated the results of the database analysis (Figure 2b). The further local cohort-based clinicopathological association analysis verified that lower TRIM50 expression was related to lymph node metastasis (*p* = .004) and advanced TNM stage (*p* = .019), irrelevant to ER as well as HER2 (Table II). The abovementioned conclusions shed light on the tumor-suppressive capacity of TRIM50.

**Table II.** Character of enrolled breast cancer patients.

Clinicopathology characteristics	TRIM50 expression		chi-square value	p-value
	highly-expressed (*n* = 22)	lowly-expressed (*n* = 23)		
Age (years)				
≤55	15	13	0.650	0.420
>55	7	10		
Size (cm)			3.780	0.151
<2	11	9		
2-5	7	13		
>5	4	1		
Lymph node metastasis			8.538	0.004**
no	18	9		
yes	4	14		
TNM stage				
I+II	20	14	5.494	0.019*
III+IV	2	9		
Estrogen receptor				
negative	4	10	3.357	0.067
positive	18	13		
Her2 receptor				
negative	13	17	1.676	0.195
positive	9	6		

*p < .05 was considered to be statistically significant, **p < .01.

### 3.2. Overexpressed TRIM50 suppressed TNBC proliferation and induced apoptosis

To validate whether TRIM50 functions as a suppressor in TNBC carcinogenesis, we detected the expression of TRIM50 in MDA-MB-231, BT549, Hs-578T TNBC cell lines, and non-transformed epithelial cell MCF-10A by WB and RT-qPCR. The results verified that the TRIM50 level was lower in MDA-MB-231, BT549, and Hs-578T TNBC cells compared with MCF-10A cells (Figures 2c,d).

Next, we constructed pcDNA3.1-TRIM50 and pcDNA3.1-vector plasmid to transfect the TNBC cell lines. TNBC transfected with pcDNA3.1-TRIM50 plasmid (pcDNA3.1-TRIM50 cohort) served as an acquisition-of-function model, while the TNBC transfected with pcDNA3.1-TRIM50 vector (vector cohort) served as a control model. TRIM50 protein expression in these stably transfected cells screened out by puromycin was detected using WB. As shown in Figure 2e, the pcDNA3.1-TRIM50 cohort exhibited significant upregulation of TRIM50 compared to the vector cohort.

**Figure 3.**
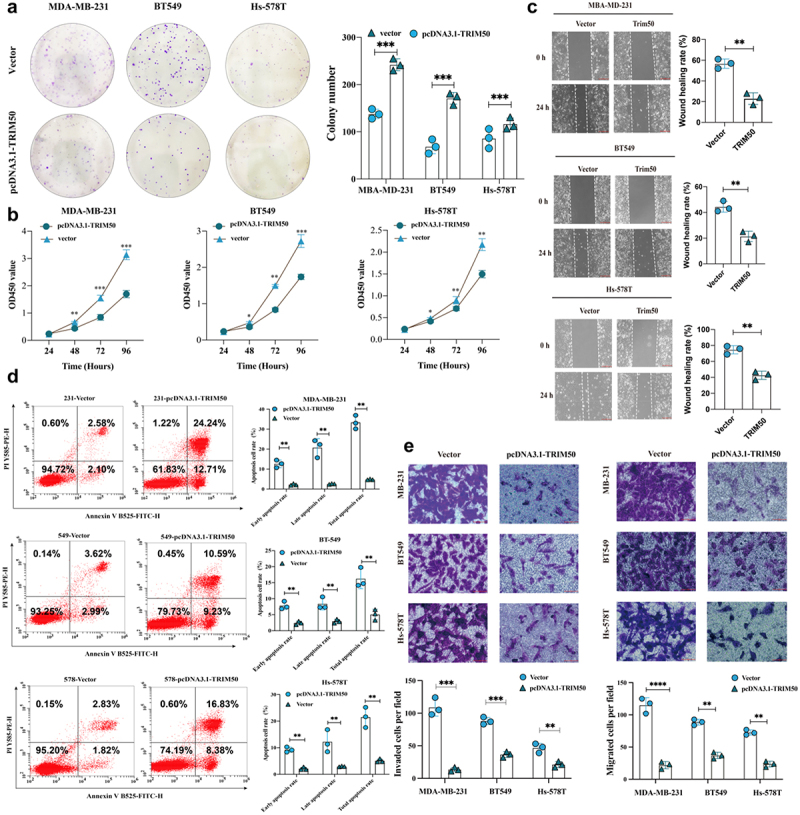
Overexpressed TRIM50 mitigated malignant phenotypes of TNBC cell lines. (a) Clone formation experiment was observed that the colony quantity aggregated in the pcDNA3.1-TRIM50 group was significantly lower than that in the vector group. (b) Proliferation rates (doublings/day) of MDA-MB 231, BT549, and Hs-578T cells were detected by cell counting kit-8 at 24, 48, 72, and 96 h respectively. And there was a statistically significant trend of difference since 48 h after inoculation. (c) The mobility of TNBC cells transfected with plasmid was detected by cell scratch experiment. Images were collected at 0 and 24 h after scratch respectively, and the scratch gap was measured quantitatively based on the standardized distance initially (10X scale: 200 µm). (d) Flow cytometry analysis showed that the percentage of apoptotic cells in the pcDNA3.1-TRIM50 group was higher than that in the vector group with statistical differences. Cells that stained positive for annexin V and negative for PI were considered early apoptotic (LR: lower right quadrant). Cells staining positive for annexin V and PI were considered late apoptotic or necrotic cells (UR: upper right quadrant). (e) Transwell migration and invasion experiments were applied to calculate the number of cells migrating or invading through the matrigel from the upper chamber to the lower chamber after 24 h incubation at a constant temperature. (10X scale: 200 µm; 40X scale: 40 µm). *p < .05, **p < .01, ***p < .001, ****p < .0001. *n* = 3 biological replicates.

To further elucidate the suppressive mechanism of TRIM50 exerting on TNBC, we performed proliferation and apoptosis-related experiments. CCK8 experiment employed to validate proliferation-relevant effect on TNBC show that TRIM50-upregulated cells exhibited weaker proliferative activities compared to wild-type cells. Similarly, colony formation assays also indicated that TRIM50 overexpression attenuated colony formation in vitro (Figures 3a,b), which suggests that TRIM50 negatively regulates cell proliferation in vitro. To determine the action of TRIM50 on cell apoptosis, Annexin-V assay was performed by flow cytometry analysis which showed that over-expressed TRIM50 mediated by plasmid transfection induced more apoptotic cell death, suggesting that the tumor-suppressor function of TRIM50 involves activation of tumor apoptosis (Figure 3d). Taken together, these findings converged to the conclusion that TRIM50 suppressed TNBC proliferation and induced apoptosis.

### 3.3. TRIM50 overexpression suppressed cell migration and invasion

To corroborate the effects of TRIM50 in TNBC migration and invasion capacity, we performed wound healing and Transwell assays. As shown in Figure 3c, the appreciable declining scratch gap could be interpreted with the understanding that TRIM50 overexpression significantly decreased the wound healing rate of TNBC cells. Similarly, TRIM50 overexpression significantly crippled the migratory and invasive capacity in vitro (Figure 3e).

### 3.4. TRIM50 interacted with SNAI1 to downregulate snail and inhibited the epithelial–mesenchymal transition (EMT) phenotype in vitro

Epithelial–mesenchymal transition phenotype was envisioned as a pivotal pathological process, and its key biomarker SNAI1-mediated downstream signaling was adversely associated with the tumor-inhibition function of TRIM50 in breast cancer cells.^33^ Snail family members are EMT-activating transcriptional factor that directly bind to the proximal CDH1 promoter to inhibit E-cadherin and reshape the intercellular adhesion^34^; It also reconstructs epithelial polarized molecules and blocks the formation of basement membrane to build a predominantly invasive-inclined tumor microenvironment.^35^

**Figure 4.**
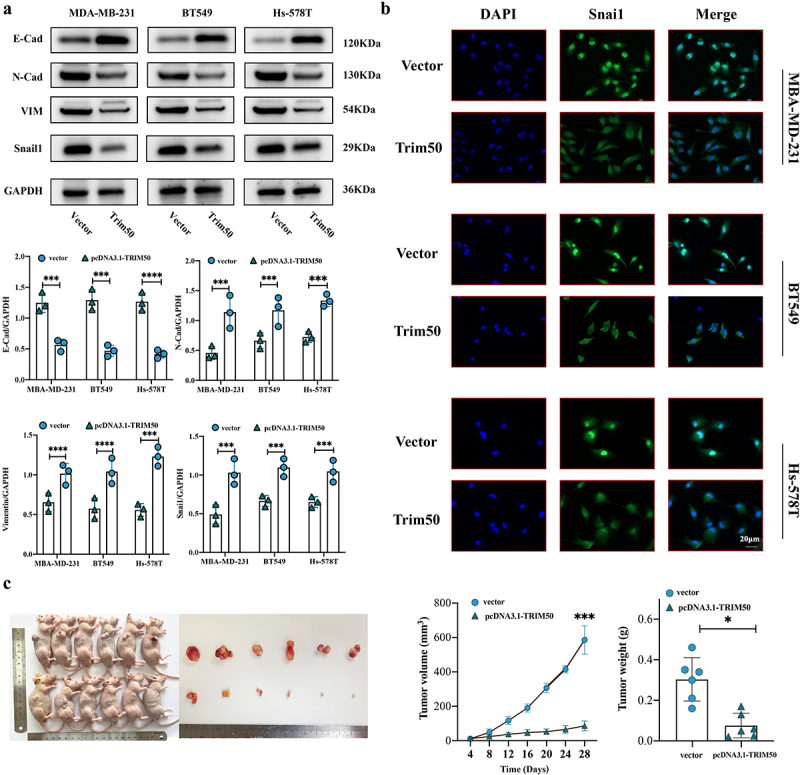
Overexpressed TRIM50 eclipsed EMT biomarkers in TNBC. (a) Upper panel: western blot analysis of the indicated emt-associated proteins in MDA-MB 231, BT549, and Hs-578T cells with the indicated plasmids transfected. Lower panel: quantification of E-cadherin, N-cadherin, Vimentin, and SNAI1 levels relative to GAPDH in western blot. (b) Immunofluorescence analysis of SNAI1 in MDA-MB 231, BT549, and Hs-578T cells with the indicated plasmids transfected; *n* = 3 biological replicates. Scale bar 40 μm. The localization and fluorescence intensity of SNAI1 (green fluorescence) in the nucleus (blue fluorescence) were detected by immunofluorescence experiment after pcDNA3.1-TRIM50 and vector plasmid were transfected respectively. Scale bar: 20 µm. (c) Overexpression of TRIM50 inhibited tumorigenesis in vivo. Growth curves of tumorigenesis in nude mice were plotted and the final tumor mass was measured. Left panel: images of tumors on day 28 after injection. *n* = 6/condition; right panel: tumor growth curves of tumorigenesis explicated in volume and weight at day 28 after injection of the above-described tumors. *n* = 6/condition. In scatter dot plots, each dot represents one mouse. Two-tailed student’s t-test was exploited.

To verify that TRIM50 participates in the EMT process of TNBC cells, Western Blotting was applied to analyze the effect of TRIM50 overexpression on EMT. The results showed a panoply of expression alterations in EMT-related biomarkers that the expression of SNAI1 was dampened in transient overexpressed TRIM50 compared to the vector group as well as N-cadherin and Vimentin, while expression of E-cadherin was markedly rising (Figure 4a). To sum up, TRIM50 may affect EMT-related phenotypic molecules by inhibiting the SNAI1-mediated EMT process, thereby inhibiting the migration ability obtained by the epithelial–mesenchymal transformation of TNBC.

We further determine the effect of TRIM50 overexpression on SNAI1 in the cells by cellular immunofluorescence. Results were observed that cells in the vector group were mostly in the mitotic phase with obvious mitotic signs (blue stained part in Figure 4b), and the expression level of SNAI1 in the nucleus was increased compared to that in the pcNDA3.1-TRIM50 group (green stained part in Figure 4b). While overexpressed TRIM50 significantly reduces the fluorescence intensity of SNAI1, the specific subcellular localization shows a particularly obvious decrease in the nucleus. The results confirm our hypothesis that TRIM50 may affect the EMT process of TNBC by inhibiting SNAI1.

### 3.5. TRIM50 attenuated TNBC proliferation in tumor-bearing mice in vivo

Through previous experiments, we have dissected that SNAI1-mediated EMT attenuation contributed to the suppressive role of TRIM50 in TNBC, thereby inhibiting the proliferation, migration, and invasion of TNBC. Thus, we established a xenograft tumor model to evaluate the effect of TRIM50 on carcinogenesis of TNBC in vivo. We selected MBA-MD-231 cells for subcutaneous injection in nude mice. Mice were assigned at random to the following experimental groups: vector group and pcDNA3.1-TRIM50 group (*n* = 6 per group).

In concert with the results evidenced by the CCK8, colony formation experiments that tumors with TRIM50 upregulation had a lower proliferation capacity, the growth curve displayed that the overexpression of TRIM50 significantly inhibited tumor volume (*p* < .001) (Figure 4c). Compared with the vector cohort, the rate of tumor formation in the pcDNA3.1-TRIM50 group dropped significantly. Then the subcutaneous tumor was separated and weighed, and we detected that tumor weight in the pcDNA3.1-TRIM50 cohort was significantly lower relative to their blank control counterparts (*p* < .05), as shown in Figure 4c. The results showed that TRIM50 could significantly suppress TNBC proliferation in vivo.

### 3.6. Immunoregulatory of TRIM50 in breast cancer

**Figure 5.**
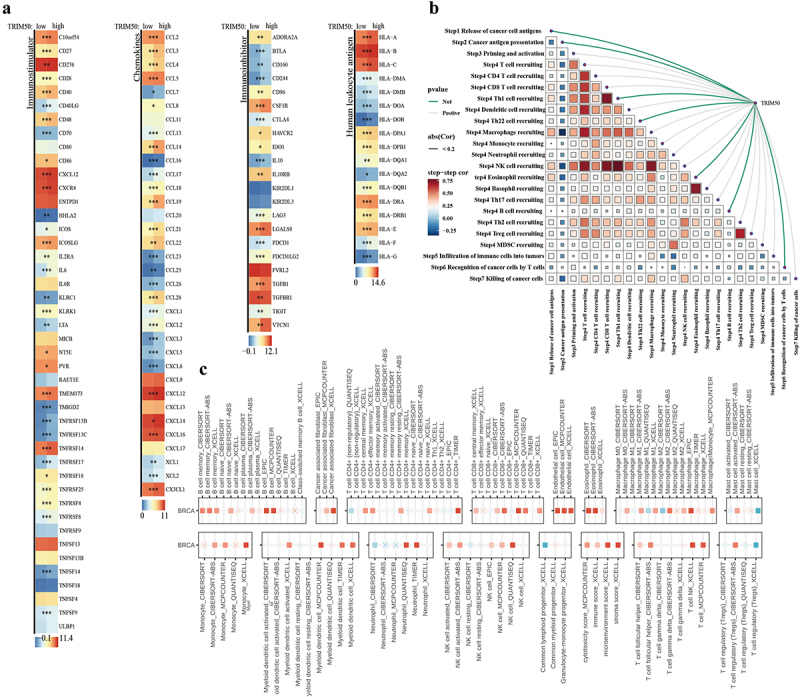
Immune characteristics of TRIM50. (a) Differences in expression of immunostimulatory genes, immunosuppressive genes, chemokines and human leukocyte antigen in high/low TRIM50 expression groups. *p < .05, **p < .01, ***p < .001. (b) Heatmap of Spearman correlation between TRIM50 expression level and tumor immune cycle activity. The red/green lines represent positive/negative correlation, the gray lines represent no significance, and the thickness of the lines represents the absolute value of the correlation coefficient. The correlation of the triangle region is represented by the color depth and size of the square. (c) Multiple algorithms evaluate the Spearman correlation between the TRIM50 and immunoinfiltrating cell contents. Multiple algorithms evaluate the Spearman correlation between the expression of TRIM50 and different immune infiltrating cells in BC.

As shown in Figure 5a, TRIM50 expression showed a remarkable positive association with

the cancer immune cycle, especially with T cell and NK cell recruitment, which proved

that TRIM50 facilitate the phenomenon of immune recruitment in tumors. TRIM50 expression trended positively with immune checkpoint expression (LAG3, CTLA4), as shown in Figure 5b. TRIM50 promoted the infiltration of CD8 T cells, Macrophage, and NK cells in BC, shaping the inflammatory tumor microenvironment (Figure 5c).

## 4. Discussion

As a new member of the TRIM family,^25,36,37^ TRIM50 was once documented as a ubiquitin ligase with proautophagic activity and a candidate tumor suppressor in hepatocellular carcinoma, ovarian cancer, and pancreatic cancer.^26,27,38^ TRIM50 could suppress the EMT event and tumorigenicity by K48-linked poly-ubiquitination of Snail protein in hepatocellular carcinoma and pancreatic cancer.^26,27^ Although the functions of TRIM50 in TNBC were not elucidated yet, these studies still provide the possibility for TRIM50 to be a tumor suppressor. As expected, TRIM50 expression in cancer tissues was significantly lower compared with paired normal breast tissues detected by western blotting and qPCR. In addition, the immunohistochemical results showed that TRIM50 distributed in the cytoplasm region of noncancer breast tissues. Besides, the correlation analysis showed that TRIM50 overexpression was significantly correlated with lymph node metastasis and senior clinical stage. To verify the effect of TRIM50 on the biological behavior of breast cancer cells, we carried out a series of experiments (CCK-8, clone formation, flow cytometry, cell scratch, and Transwell experiment). The results showed that overexpression of TRIM50 significantly inhibited the malignant biological behavior of TNBC such as proliferation, migration, and invasion. The above data confirmed that TRIM50 plays an anti-tumor role in TNBC, and the low expression or deletion of TRIM50 in breast cancer may lead to an increase in tumor malignancy.

**Figure 6.**
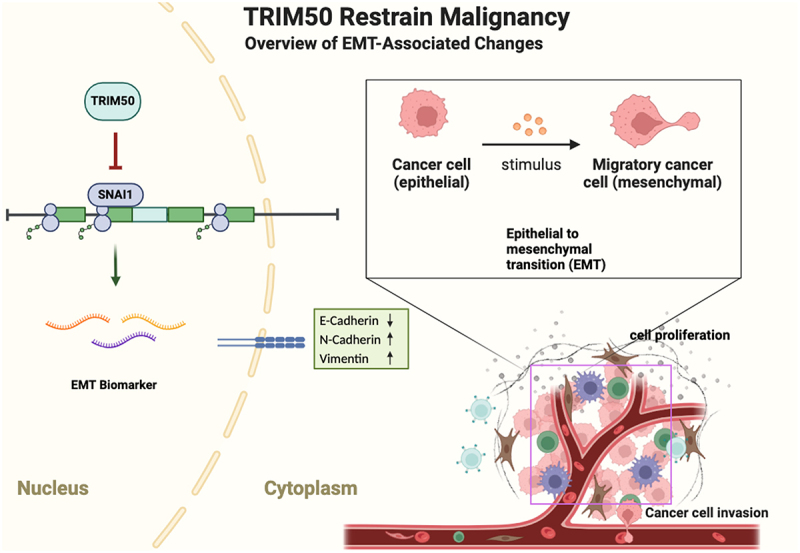
The schematic diagram illustrated that TRIM50 suppresses TNBC progression by regulating EMT process.

As one of the key mechanisms of tumor progression, the EMT phenotype is manifested in the destruction of cell contact and the enhancement of deformation, movement, and migration, leading the migration from the primary site to distant metastasis. Studies have confirmed that SNAI1 could regulate the downstream EMT phenotypic markers of epithelial and mesenchymal cells and is deemed as an important EMT-induced transcription factor. Based on the study of TRIM50 in liver and pancreatic cancer, it was found that TRIM50 blocked EMT by targeting SNAI1 and down-regulating its expression. TNBC overexpressed with TRIM50 disclosed a remarkable decrease in the levels of N-cadherin, vimentin, and SNAI1 by western blots, while increasing E-cadherin level, as shown in Figure 6. It was also observed by immunofluorescence staining that TRIM50 overexpression attenuated the nuclear localization of SNAI1. Therefore, this study assumed that TRIM50 could adversely regulate SNAI1 level in TNBC. The xenograft experiment further confirmed that TRIM50 could inhibit the proliferation of TNBC in vivo. To sum up, these findings preliminarily explored and confirmed the effect of TRIM50 on inhibiting proliferation and invasion in TNBC, providing a novel insight into the treatment of TNBC.

A variety of transcriptional factors (TFs) are cruxes for interpreting signals transmitted from stimuli to transcription factors in the EMT process. These EMT-TFs are associated with the maintenance of initial migration phenotype and invasive cell behavior of several primary tumors. As a key regulator of EMT, SNAI1 plays a significant role in tumor invasion and metastasis. Studies have shown that SNAI1 is more expressed in TNBC than other EMT-TFs, and evidenced by SNAI1 expression in TNBC showed an about 10-fold increase (*p* value = 0.008) compared with ZEB1 and TWIST at transcriptional level. Besides, SNAI1 is assumed as one of the most important factors concerning tumor invasiveness, while the down-regulation of ZEB-1 and TWIST is associated with tumor progression and poor prognosis.

Molecular events mediated by SNAI1 are interesting targets for treatment, especially for drug-resistant tumors. It is reported that the molecular events during tumor progression are reversible, such as loss and gain of E-cadherin in primary lesion and distant metastasis, which could be explained by post-translational modification events (PTM), involving hypermethylation and deacetylation of DNA. Although directly targeting SNAI1 seems not successful, the identification of PTM inhibitors toward SNAI1 has tremendous potential, and thus it is a high priority in the future development of cancer therapy. In addition, the identification of PTM events toward SNAI1 in primary tumors is quietly critical, because these acknowledgments will help to better predict genotypes that are more likely to follow invasive processes and identify those patients who are prone to develop metastasis. Deubiquitin enzyme (DUB) is known to counteract the degradation of SNAI1. Independent studies have shown that DUB3 interacted with SNAI1 and stabilized SNAI1 level. DUB3 activation mediated by CDK4 and 6 is very important for the deubiquitination and stabilization of SNAI1. At the transcriptional level, EMT and transfer-inducing factors such as TGF-β, TNF-α, and hypoxia affect the phenotype of EMT by regulating EMT-TF including SNAI1. In view of the important role of SNAI1 in promoting cancer progression, targeting SNAI1 will be an attractive anti-cancer therapy. CYD19 compound weakens tumor invasion and metastasis by reversing SNAI1-driven EMT, which suggests that pharmacological interference on SNAI1 epigenetic modification may take effect in patients.

TNBC was known as an immune-cold tumor with fewer immune infiltration, while EMT-TF has been shown to regulate the expression of several inhibitory immune checkpoints. Therein, SNAI1 displayed a closer relationship with immune microenvironment inhibition. Recent studies have found that SNAI1 up-regulated CD73 by directly binding to the proximal promoter of CD73. SNAI1-dependent upregulation of CD73 led to increased production and release of extracellular adenosine in TNBC cells and helped to enhance the immunosuppressive property of TNBC.

TRIM proteins have been widely recognized as pro-inflammatory modulators, as well as important antiviral restriction factors. In this report, the results confirmed that the upregulation of TRIM50 formed an inflammatory tumor microenvironment and activated the immune response in BC. Specifically, CD8+ T cells, dendritic cells, macrophages, NK cells, and Th1 cells in the tumor microenvironment were effectively recruited through activating TRIM50. In addition, it was found that chemokine promoted CD8+ T cell aggregation and translocation to the tumor site, where CD8+ T cells acting on tumor cells could achieve survival progression. In our analysis, most chemokines showed a trend of upregulation in the TRIM50-High group. These effects of TRIM50 on immune infiltration cells and cytokines could significantly influence the metastatic potential of tumors by creating an inflammatory tumor microenvironment.

A majority of studies focused on the E3 ubiquitin ligase activity of TRIM50 in carcinogenesis, immunity, and autophagy. TRIM50 has been proved to achieve its tumor inhibition function in a variety of cancers through ubiquitination mediated by its RING domain. For example, Gu et al. found that TRIM50 inhibited glycolysis pathway of gastric cancer cells (GCs) through ubiquitylation and degradation of PGK1, thus directly inhibiting the proliferation of GCs. In turn, decreased lactic acid weakened the M2 polarization of tumor-associated macrophages (TAMs) and inhibited the invasion and migration of GCs indirectly. In the process of autophagy regulation, TRIM50 supported the signal transduction activation and multi-target recognition. It has been proved that HDAC6-mediated deacetylation of TRIM50 promoted its ubiquitination of Beclin-1. In addition to post-translational modification of autophagy-related proteins such as Beclin-1, TRIM50 could cooperate with p62 scaffold protein to selectively recognize autophagy cargoes and promote the formation and autophagy clearance of poly-ubiquitin proteins by interacting with HDAC6. In addition, TRIM50 also functioned in anti-viral immunity in a way distinct from its conventional E3 ligase function. TRIM50 induced NLRP3 oligomerization through its RING domain independent of the RING-type ubiquitylation activity, and in turn activated NLRP3 inflammasome. The role of NLRP3 inflammasome was elucidated in host defense against pathogens, and their abnormal activation correlated with a variety of diseases, including cardiovascular, diabetic, and neurodegenerative diseases. Thus, the development of novel inhibitors for NLRP3 inflammasome targeting at TRIM50 could be of great significance.

However, the current research still has some limitations. First, this study only preliminarily confirmed that TRIM50 was adversely correlated with the nuclear expression of the transcription factor SNAI1 and has not fully elucidated the regulatory mechanism of TRIM50 on SNAI1 in breast cancer. Second, based on the ubiquitination capacity in TRIM family members, it is necessary to detect whether TRIM50 directly targets SNAI1 through its ubiquitination sites and detect the ubiquitination level. Third, this study needs a larger sample to test the predictive effect of TRIM50 as a biomarker in TNBC.

## 5. Conclusion

In conclusion, we demonstrated that TRIM50 suppressed TNBC proliferation and invasion by regulating the EMT process in vitro and in vivo. Clinical evaluation indicated that TRIM50 was associated with the clinicopathological characteristics of malignancy. Together, our findings presented a potential therapeutic target and prognostic indicator of TRIM50 in breast cancer. Inspired by the Deep-going exploitation on the precise mechanisms of TRIM family will allow for targeting TRIM in TNBC treatment. TRIM50 warranted further researches to provide bright insight into its translational values in TNBC.

## Data Availability

The data that support the findings of this study are available from the corresponding author, LXJ, upon reasonable request.

## Animal studies

The abovementioned animal experiments were approved by the Animal Experimentation Ethics Committee of Ningbo No.2 hospital (approval No. wydw2021-0063)

## Abbreviations

AJCC: American Joint Committee on Cancer
BC: breast cancer
ER: estrogen receptor
HER2: human epidermal growth factor receptor-2
EMT: epithelial-mesenchymal transition
FBS: fetal bovine serum
GEO: gene expression omnibus
IF: immunofluorescence
IHC: immunohistochemistry
PBS: phosphate-buffered saline
PR: progesterone receptor
RT-qPCR: real-time quantitative polymerase chain reaction
SPF: specific pathogen-free
TNBC: triple-negative breast cancer
TRIM50: tripartite motif-containing protein 50
UB: ubiquitin
WB: western blotting
