# Correction

**DOI:** 10.1080/15384047.2025.2585668

**Published:** 2025-11-06

**Article title:** Tripartite motif-containing protein 50 suppresses triple-negative breast cancer progression by regulating the epithelial–mesenchymal transition

**Authors:** Danxiang Chen, Jing Jiang, Wei Zhang, Xinlin Li, Qidong Ge, Xia Liu, and Xujun Li

**Journal:** *Cancer Biology & Therapy*

**Bibliometrics:** Volume 25, Number 1, pages 1−14

**DOI:** https://doi.org/10.1080/15384047.2024.2427410

This article was first published with errors:

In the original version of this manuscript, an error was found in Figure 3c.

The article has been republished with the errors corrected.

